# TERTmonitor Efficacy and Performance in Detecting Mutations by Droplet Digital PCR

**DOI:** 10.3390/genes15111424

**Published:** 2024-10-31

**Authors:** Mafalda Bessa-Gonçalves, João Paulo Brás, Tito Teles Jesus, Hugo Prazeres, Paula Soares, João Vinagre

**Affiliations:** 1 U-Monitor Lda, 4200-135 Porto, Portugal; mafalda.goncalves@uromonitor.com (M.B.-G.); joao.bras@uromonitor.com (J.P.B.); hprazeres@ipatimup.pt (H.P.); jvinagre@ipatimup.pt (J.V.); 2 Instituto de Investigação e Inovação em Saúde (i3S), Universidade do Porto, 4200-135 Porto, Portugal; tjesus@ipatimup.pt; 3 Instituto de Patologia e Imunologia Molecular da Universidade do Porto (Ipatimup), 4200-135 Porto, Portugal; 4 Faculdade de Medicina, Universidade do Porto (FMUP), 4200-319 Porto, Portugal

**Keywords:** telomerase promoter, real-time qPCR, ddPCR, TERTmonitor, mutations

## Abstract

Background: The screening of TERT promoter (*TERTp*) mutations is essential in cancer research and diagnostics, due to its prevalence in tumours associated with low self-renewal rates. TERTmonitor is a diagnosis kit primarily designed for real-time qPCR qualitative detection of −124C>T and −146C>T *TERTp* mutations, which are highly prevalent in several malignancies, particularly in bladder carcinoma. Objective: This study aims to investigate TERTmonitor performance in droplet digital PCR (ddPCR) in urine samples from bladder cancer patients. Methods: A total of 45 urine samples were examined by real-time qPCR and ddPCR techniques, and their performances were compared. Results: TERTmonitor had similar performance in both real-time qPCR and ddPCR platforms. Specifically, the methods exhibited a concordance rate of 95.45% and 90% for −124C>T and −146C>T mutations, respectively. Importantly, an enhanced sensitivity in certain scenarios was exhibited by ddPCR when compared to real-time qPCR, detecting mutations that the latter failed to identify in approximately 4.55% and 10% of the samples for −124C>T and −146C>T mutations, respectively. This enhanced sensitivity of ddPCR was particularly evident in samples with low-frequency mutations. Conclusions: The findings highlight the usefulness of TERTmonitor for cancer surveillance either in real-time qPCR or ddPCR platforms.

## 1. Introduction

The screening of telomerase promoter (*TERTp*) mutations holds paramount importance in the realm of cancer diagnostics and research due to their pervasive presence in tumours derived from cells with low self-renewal rates [1,2]. The most prevalent *TERTp* mutations represent critical genetic alterations across various cancer types, significantly influencing the pathogenesis and clinical outcomes of affected individuals. Unlike the infrequent occurrence of mutations in the coding region of the *TERT* gene, mutations in the *TERTp* are notably frequent in some human cancers [3]. The primary sites affected by *TERTp* mutations are positioned at 124 and 146 base pairs upstream from the translational start site, resulting in C to T transitions (−124C>T and −146C>T) [4,5]. These alterations have been identified as key contributors to the molecular characteristics of various malignancies, including glioblastoma [6,7,8], melanoma (where they were first identified) [4,5], urothelial bladder [6,9,10,11], and thyroid carcinoma [12,13,14].

Various molecular techniques are employed for *TERTp* mutation detection, like Sanger and exome sequencing, with real-time quantitative polymerase chain reaction (real-time qPCR) [15,16,17,18] and droplet digital polymerase chain reaction (ddPCR) [19,20] representing two prominent approaches. While real-time qPCR provides semi-quantitative results, ddPCR offers absolute quantification by partitioning DNA into droplets, ensuring precision in quantifying target DNA. In technical terms, real-time qPCR’s advantage lies in real-time monitoring, broad dynamic range, and high sensitivity, but it faces challenges in detecting mutations in low-abundance samples and is susceptible to inhibitors, potentially affecting result accuracy [21]. In contrast, ddPCR is less prone to inhibitors, providing higher accuracy for rare mutations, but it has a lower dynamic range, making it less suitable for high-abundance targets [22].

TERTmonitor is an ultrasensitive and specific kit that employs proprietary technology for detecting −124C>T and −146C>T *TERTp* mutations. It is an adaptation of the commercially available real-time qPCR kit Uromonitor^®^ that was designed for the genotyping of *TERTp*, *FGFR3*, and *KRAS* hotspot mutations in non-muscle invasive bladder cancer patients under surveillance [16,18,23,24]. TERTmonitor real-time qPCR performance was recently tested in gliomas [17]. It performed with 97.8% accuracy against the standard analysis method, Sanger DNA sequencing, strengthening the potential of this tool for *TERTp* genotyping. *TERTp* mutations detected in urine samples show great promise as non-invasive biomarkers for bladder cancer diagnosis, monitoring, and prognosis. These alterations can be detected in urine up to 10 years prior to a clinical diagnosis, making them valuable for early detection [25,26]. There is a high concordance between *TERTp* mutations detected in urine and their respective tumour tissue counterpart samples [25]. In a bladder cancer surveillance setting, *TERTp* mutations in urinary cell free DNA were associated with tumour recurrence following transurethral surgery for non-muscle invasive bladder cancer, suggesting their potential prognostic markers capacity and to guide follow-up strategies. The high frequency of *TERTp* mutations and across different stages of bladder cancer (ranging from 60–80%) makes them particularly useful diagnostic markers [2]. Importantly, simple and sensitive detection methods like ddPCR allow for the identification of these mutations in urine samples with promising diagnostic accuracy, especially for primary bladder cancer detection. This non-invasive approach could potentially reduce the need for frequent invasive procedures like cystoscopy in bladder cancer monitoring.

In the present study, our goal was to explore TERTmonitor applicability and performance in a ddPCR setup. TERTmonitor is adapted for use in non-invasive settings, and in the present study, we apply the ddPCR test to filtered urine samples from bladder cancer patients to ensure accurate validation and a thorough assessment of the test’s performance in comparison with the real-time qPCR.

## 2. Materials and Methods

### 2.1. Clinical Samples/Specimens

All procedures described in this study involved the repurposing of anonymized secondary data collected from two previously conducted clinical studies. This research did not involve the direct participation of human subjects, nor did it collect sensitive or identifiable information. Additionally, there were no clinical interventions, and no new risks were posed to participants. Urine samples were collected from patients previously diagnosed with non-muscle invasive bladder cancer (NMIBC), who were undergoing surveillance of bladder cancer as part of the routine clinical monitoring. During their regular follow-up cystoscopy appointments, all patients were requested to provide urine samples (n = 45) before examination. Following collection, a minimum of 10 mL of urine was filtered using the “Urokit1—Urine filtering kit” unit of Uromonitor^®^ (U-Monitor, Porto, Portugal), following the manufacturer’s instructions. Filters were then stored at 4 °C up to 2 weeks before DNA extraction.

Inclusion criteria were the following: patients must be at least 18 years of age; have a history of primary non-muscle invasive bladder cancer (NMIBC) in any risk group, having received any intravesical adjuvant treatment within the last 3 months to 2 years, and undergoing regular cystoscopic surveillance; be able to provide a minimum of 10 mL of urine prior to cystoscopy, with an additional 10 mL collected for cytology; and be able to give informed consent. Exclusion criteria: patients undergoing cystoscopy for a different diagnosis; those with a previous diagnosis of muscle-invasive bladder cancer (MIBC) or upper tract urothelial carcinoma (UTUC); patients planning to undergo radical cystectomy or chemotherapy/radiation for urinary cancer (UC); those unable to provide the minimum amount of urine needed for testing; patients at risk for additional relevant findings; and patients providing inadequate material for Uromonitor^®^ testing.

### 2.2. DNA Extraction

DNA extraction was performed using the “Urokit2—DNA extraction and purification kit” unit of Uromonitor^®^, following the manufacturer’s instructions [23]. Briefly, exfoliated bladder cells trapped in the filter were lysed by adding 400 μL of cell lysis buffer into it and by incubating the solution at 60 °C for 30 min with agitation. Proteinase K was then added, and the mixture underwent an additional 10-min incubation at 60 °C. After introducing a normalizing solution and vortexing for 5 s, the lysate mixture was transferred to a spin column and centrifuged. The flow-through was discarded, and the spin column underwent centrifugation to dry the matrix. After that, a two-step washing centrifugation was performed. The spin column was finally moved to an elution tube, and an elution buffer was added. After incubation, the mixture was centrifuged to collect the eluate containing the DNA. The extracted DNA was then quantified in a Nanodrop 1000 (Supplementary Table S1) and stored at −20 °C for subsequent Real-Time qPCR and ddPCR analysis.

### 2.3. Real-Time qPCR

Real-time qPCR was employed using the proprietary compositions of TERTmonitor for detecting −124C>T and −146C>T mutations in *TERTp*, as described in [17]. The analysis was conducted on a StepOnePlus™ qPCR instrument (ThermoFisher Scientific, Waltham, MA, USA). Briefly, two independent multiplex reactions were prepared for each mutation, using FAM for mutation (*TERTp* −124C>T and *TERTp* −146C>T) and HEX for wildtype (wt) (*TERTp* −124 wt and *TERTp* −146 wt). The preparation of mixes followed the manufacturer’s guidelines, incorporating components such as the master mix, specific primers, and probes for detecting hotspot mutations, along with corresponding wildtype controls for quality assessment. TERTmonitor uses proprietary technology for its primers and probes with patented methodology, and specific details are not disclosed. This technology provides exceptional sensitivity and specificity in targeting the designated sequences. Plates were set up by adding 9 μL of mix and 1 μL of DNA. Multiplex qPCR runs comprised a cycle at 95 °C for 3 min, followed by 50 cycles at 95 °C for 20 s and 68 °C (*TERTp* −124)/64 °C (*TERTp* −146) for 45 s.

### 2.4. Droplet Digital PCR (ddPCR)

Workflow procedures for ddPCR were performed according to the manufacturer’s instructions for the QX200™ system (BioRad Laboratories, Hercules, CA, USA). The reaction mixture had a volume of 22 μL, consisting of a 20 uL mixture of ddPCR Supermix 4× (BioRad Laboratories), TERTmonitor’s proprietary primer/probes compositions, and 2 μL of DNA (varying concentration); an identical quantity of DNA was used as the template for both real-time qPCR and ddPCR. The partitioning of samples into nanoliter-sized droplets was accomplished using a QX200™ Droplet Generator (Bio-Rad Laboratories), employing single-use DG8 cartridges and Droplet Generation Oil (Bio-Rad Laboratories). Each reaction mix (20 µL) was loaded onto the cartridge, and an emulsion was formed with 70 µL of oil. Resultant droplets were manually transferred using a multichannel pipet to a ddPCR™ 96-well PCR plate (Bio-Rad Laboratories), subsequently sealed with a foil cover through heat-sealing [19,20]. Next, the droplets underwent thermocycling using a PTC Tempo 96 Thermal Cycler (BioRad Laboratories) with a ramp rate of 2 °C/s at each step. The process was initiated with an initial enzyme activation step at 95 °C for 10 min, followed by 50 cycles of denaturation at 95 °C for 30 s, primer annealing at an optimized temperature (68 °C for −124C>T and 64 °C for −146C>T) for 1 min, followed by 98 °C for 10 s (enzyme deactivation). Post-amplification, the droplets were promptly analysed using Bio-Rad’s Droplet Reader (Bio-Rad Laboratories) [27].

### 2.5. Data Analysis

Analysis of real-time qPCR data was performed using the StepOne™ Software v2.3 (ThermoFisher Scientific). Multicomponent and amplification signals in FAM and HEX channels were acquired and analysed applying the thresholds recommended by the manufacturer. If at least one of the screened alterations provided a positive result in replicates, the test was considered positive. For inconclusive signals in replicates, a novel independent reaction was generated; if two positive signals were obtained in different reactions, a positive result was considered. Positive and negative control samples were included for the assay’s validity. Real-time qPCR data are presented in mutant (Ct mut) and wild-type Ct (Ct wt) values (Supplementary Table S2). Valid wild-type (WT) Ct values for this assay range between 25–40, while mutant (Mut) Ct values are considered valid below 45.

Analysis of ddPCR data was performed using the QuantaSoft™ Software v2.2 (BioRad Laboratories) applying manual threshold. Data were exported from the QuantaSoft™ Software to Microsoft Excel. Samples with a limited count of measured droplets (<10,000) were excluded from the analysis. The exclusion of samples with <10,000 droplets ensures the accuracy and reliability of the quantification. Each droplet in ddPCR serves as an independent reaction chamber, and having fewer than 10,000 droplets reduces the statistical power of the assay, increasing the risk of sampling errors and unreliable detection of low-abundance targets. With fewer droplets, the precision of distinguishing between positive and negative droplets diminishes, potentially leading to biased or inaccurate results. By applying this exclusion criterion, the analysis maintains a necessary threshold for confidence in quantification, providing more consistent and reproducible data across samples. The number of accepted droplets, mutant-positive ((+) mut), mutant-negative ((−) mut), wild-type-positive ((+) wt), wild-type-negative ((+) wt), as well as the fractional abundance (FA) (i.e., proportion of mutant DNA alleles present in a sample containing both mutant and wild-type DNA) are presented in Supplementary Table S3. The determination of positive and negative samples was established based on FA, where values below 1 were categorized as wild-type (WT), and those exceeding this threshold were identified as positive (MUT) for −124C>T and −146C>T assays.

Linear regression analysis was conducted using GraphPad Prism 9 to investigate the relationship between Ct values (RT-PCR) and log-transformed copy concentrations (copies/µL, ddPCR) for both WT and Mutstrains of the *TERTp* mutations −124C>T and −146C>T. Log transformation was applied to the ddPCR copy concentrations to ensure a more linear relationship with the Ct values. For each dataset, regression equations, goodness-of-fit parameters (R^2^), and the statistical significance of the slopes were calculated to assess the strength and nature of the correlations. Samples that lacked either Real-time qPCR or ddPCR values were excluded from the correlation analysis to ensure data accuracy and consistency across all comparisons.

## 3. Results

### 3.1. Performance of TERTmonitor in Detecting TERTp Mutations in Urinary DNA Assessed Through Real-Time qPCR and ddPCR

DNA extracted from filtered urine samples of 45 patients was analysed for *TERTp* hotspot mutations at positions 1,295,228 and 1,295,250 on chromosome 5, located at −124 and −146 base pairs upstream from the *TERTp* ATG start codon, respectively. TERTmonitor performance was assessed via real-time qPCR and ddPCR.

In the real-time qPCR analysis, among the 45 screened samples, 30 featured *TERTp* −124C>T mutations, accounting for 66.7% of the cases, while 9 exhibited *TERTp* −146C>T mutations, representing 20.0% of the cases. Moreover, 1 sample (2.2%) displayed both mutations (Supplementary Table S2). Following this, the 45 samples underwent additional scrutiny through ddPCR analysis.

Among the 45 samples, and based on the previously defined criteria, 1 sample was excluded from the −124C>T analysis and 5 were excluded from the −146C>T analysis since there was a count of less than 10,000 droplets. The results revealed 31 samples bearing −124C>T mutations (70.5% of the cases) and 11 samples with −146C>T mutations, representing 27.5% of the cases. Notably, within these samples, 4 exhibited double positivity for both mutations (Supplementary Table S3).

### 3.2. Comparative Analysis of TERTmonitor Performance in Real-Time qPCR Versus ddPCR

In the comparative examination (head-to-head, H2H) of the efficacy of TERTmonitor in identifying *TERTp* mutations by real-time qPCR and ddPCR, a systematic categorization of detection events into discernible scenarios was undertaken. Two main scenarios were considered for the comparative evaluation: (1) positive detections by both real-time qPCR and ddPCR ((+/+) for mutated samples and (−/−) for wild-type samples); and (2) negative detection by real-time qPCR and positive detection by ddPCR (−/+). Out of the 45 samples, only those for which analysis could be conducted using both techniques were included in the comparative assessment. The outcomes of this comprehensive comparison are presented in Table 1.

**Figure 1:**
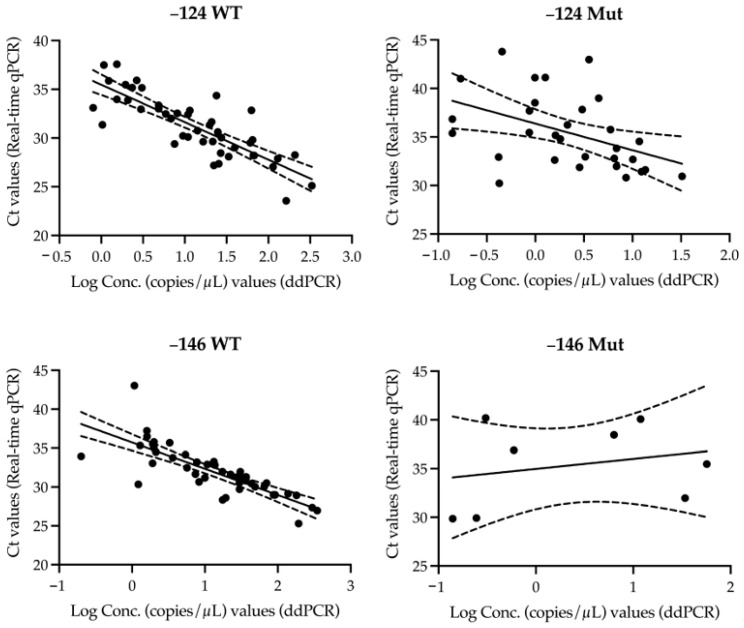
Correlation between Ct values (real-time qPCR) and log-transformed concentration (copies/µL, ddPCR) for *TERTp* mutations (−124C>T and −146C>T). (**Top left**) Ct values obtained by RT-PCR and concentration values measured by ddPCR show a significant correlation in the −124 WT strain (R^2^ = 0.68, slope = −3.849, *p* < 0.0001) (**Top right**) In contrast, the −124 Mut strain exhibits a weaker correlation (R^2^ = 0.20, slope = −2.740, *p* = 0.0132), with increased variability at lower copy numbers. (**Bottom left**) The −146 WT strain also demonstrates a significant negative correlation (R^2^ = 0.64, slope = −3.377, *p* < 0.0001) while the −146 Mut strain (**Bottom right**) showed a weak positive correlation (R^2^ = 0.06, slope = 1.026, *p* = 0.5531). Dashed lines represent the 95% confidence intervals for the regression lines.

The analysis of Ct values from real-time qPCR concerning ddPCR copy concentrations revealed significant differences between wild-type and mutant samples, underscoring the varying effectiveness of each assay (Figure 1). The −124 WT assay demonstrated the strongest performance with a regression slope of −3.849 and an R^2^ value of 0.68 (*p* < 0.0001). This strong negative correlation between log-transformed copy concentrations and Ct values indicates highly efficient amplification across a broad range of nucleic acid concentrations, reaffirming the assay’s reliability for accurate detection in both research and clinical settings. In contrast, the amplification of −124 Mut samples exhibited greater variability, reflected by a slope of −2.740 and an R^2^ value of 0.20 (*p* = 0.0132). The weaker correlation suggests that this assay does not have the same performance regarding different concentrations. The performance of the −146 WT assay was also robust, with a slope of −3.377 and an R^2^ value of 0.64 (*p* < 0.0001), showing a strong negative correlation between log-transformed copy concentrations and Ct values. This performance closely mirrors that of the −124 WT assay. Again, the −146 Mut assay displayed a weaker correlation, with a slope of 1.026 and an R^2^ value of 0.06 (*p* = 0.5531), indicating that this assay presents a lower efficacy at capturing the relationship between log-transformed copy concentrations and Ct values. The non-significant slope further highlights its limitations, particularly in detecting low-abundance targets. Overall, the insights from both real-time qPCR and ddPCR emphasise the need to utilise ddPCR only if enhanced detection capabilities are required, particularly in capturing very low-abundance targets and improving our understanding of the dynamics of mutant variants or minimal residual copies.

**Figure 2:**
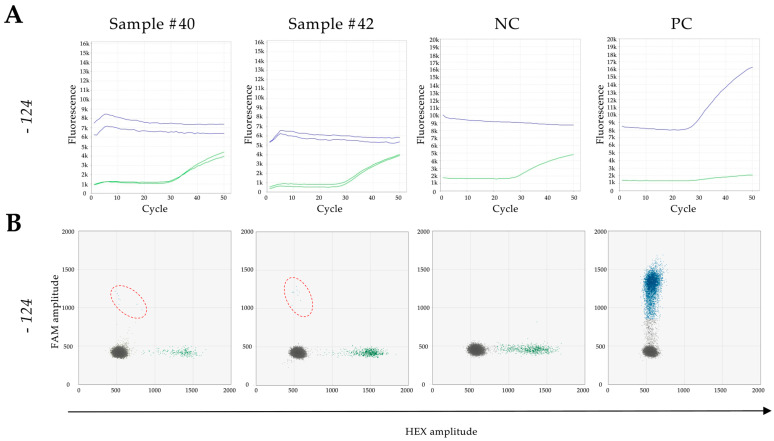
TERTmonitor real-time qPCR and ddPCR analysis of −124C>T mutation—incongruous samples. (**A**) Real-time qPCR runs were performed with 2 replicates for each sample (Sample #40 and #42). The −124C>T mutation (mut, blue, FAM) was not detected in Sample #40 or Sample #42. These samples only showed the wild-type sequence (wt, green, HEX). This observation was consistent with negative (NC) and positive controls (PC) (1 replicate). (**B**) Two-dimensional (2D) amplitude plots were generated for ddPCR analysis. Mutation-positive droplets are represented in blue (FAM positive), while droplets with the wild-type sequence are marked in green (HEX positive). Droplets with no template are presented in black. Although the −124C>T mutation was not detected in real-time qPCR, mutation-positive droplets (blue, FAM positive) were observed in both samples via ddPCR. These mutation-positive droplets are highlighted with red dashed circles. This observation aligns with the analysis of the corresponding negative (NC) and positive control (PC).

Among the 44 samples analysed for the −124C>T mutation, 42 (95.45%) exhibited concordant detections through both real-time qPCR and ddPCR. Interestingly, 2 samples (4.55%) demonstrated positivity exclusively in the ddPCR analysis (Figure 2).

**Figure 3:**
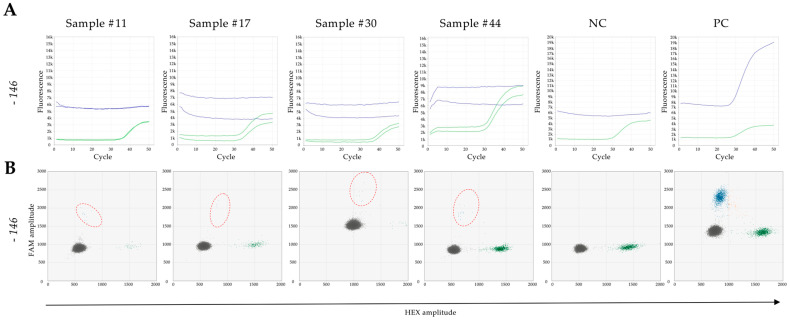
TERTmonitor real-time qPCR and ddPCR analysis of the −146C>T mutation—incongruous samples. (**A**) Real-time qPCR runs were performed with 2 replicates each sample (Sample #11, #17, #30 and #44). The −146C>T mutation (mut, blue, FAM) was not detected in Sample #11, Sample #17, Sample #30 and Sample #44 (wt, green, HEX). These samples only showed the wild-type sequence (wt, green, HEX). This observation was consistent with the negative (NC) and positive control (PC) (1 replicate). (**B**) Two-dimensional (2D) amplitude plots were generated for ddPCR analysis. Mutation-positive droplets are represented in blue (FAM positive), while droplets with the wild-type sequence are marked in green (HEX positive). Double positive droplets appear in orange (FAM and HEX positive), while droplets with no template are presented in black. Mutation-positive droplets are highlighter with red dashed circles. Although the −146C>T mutation was not detected in real-time qPCR, mutation positive droplets were observed via ddPCR. This observation aligns with the analysis of the corresponding negative (NC) and positive controls (PC).

Regarding the −146C>T mutation, in 40 valid samples, 36 (90%) showed consistent detections with both real-time qPCR and ddPCR. Additionally, 4 samples (10%) revealed positivity solely in the ddPCR assay (Figure 3). These results indicate comparable performances between Real-time qPCR and ddPCR in detecting TERTp mutations. However, ddPCR appears to provide TERTmonitor with enhanced sensitivity in the detection of mutations present in concentrations below the detection limit in real-time qPCR.

## 4. Discussion

In this study, we specifically explored and validated the use of TERTmonitor with cell-free DNA in voided urine from non-muscle invasive bladder cancer samples, using real-time qPCR and ddPCR. Previous research has demonstrated its applicability to fresh tissue and formalin-fixed paraffin-embedded (FFPE) tissue samples [17].

Urine samples stand out as an optimal reservoir for studying mutations originating in urogenital tumours. On one side, samples can be obtained by non-invasive methods, which is highly comfortable for the patient, and on the other side, it is a suitable material given the abundance of exfoliated cells from the urinary tract epithelium [28,29]. The challenge arises from the dilution of tumour-derived cells in patients’ urine, where normal epithelial cells prevail, resulting in a very low abundance of tumour cells. Consequently, the detection of these urinary mutations necessitates the application of highly sensitive methods [30,31,32]. Our results demonstrated, for the first time, that TERTmonitor exhibits comparative performance in detecting *TERTp* −124C>T and −146C>T mutations using both real-time qPCR and ddPCR setups in DNA extracted from urine-filtered samples.

Our analysis focused on two distinct scenarios, allowing for a nuanced understanding of the concordance and discrepancies between the two techniques. In the assessment of the −124C>T mutation, a high level of concordance was observed, with 95.45% of samples showing positive detections through both real-time qPCR and ddPCR. This robust agreement underscores the reliability of both methods in identifying specifically this frequent mutation. Furthermore, the analysis of the −146C>T mutation revealed an equally high concordance, with 90% of samples exhibiting consistent detections with both real-time qPCR and ddPCR. However, the identification of mutations exclusively in ddPCR in 4.55% of the samples highlights the importance of considering the strengths and limitations of each technique. The small proportion of samples (4) showing exclusive positivity in the ddPCR assay suggests a potential superiority in certain scenarios. This finding supports the idea that ddPCR is often considered more sensitive in detecting mutations at low frequencies due to its ability to partition the reaction into thousands of droplets, each serving as an individual reaction chamber [33]. In contrast, real-time qPCR may have limitations in detecting less abundant mutations, especially when they coexist with a predominant wild-type population [34]. The negative detection by real-time qPCR and positive detection by ddPCR in specific samples might suggest the presence of low-frequency mutations that go undetected by the less sensitive real-time qPCR.

Importantly, the careful exclusion of samples with inadequate droplet counts underscores the commitment to methodological rigour, essential for maintaining the integrity of the ddPCR analysis, particularly in the context of challenges posed by the rain effect. This phenomenon, characterized by scattered droplets between positive and negative clusters, complicates the interpretation of mutation results by introducing noise, particularly in low-frequency mutations. Previous studies reporting the efficacy of ddPCR assays in identifying *TERTp* mutations in cell-free DNA from urine [31,33,34,35] have also highlighted the challenges posed by the rain effect, noting its potential to overestimate mutation abundance in cases of low-input or subtle mutation frequencies. In our study, while high concordance was observed between real-time qPCR and ddPCR in detecting *TERTp* mutations, the rain effect’s influence on ddPCR results requires careful consideration.

Interestingly, in certain samples, ddPCR detected additional mutations that were not identified by real-time qPCR even though real-time qPCR had already detected mutations in those cases. This underscores the superior sensitivity of ddPCR, particularly in samples with low mutation abundance. Despite the low fractional abundance, ddPCR reliably distinguished negative controls without producing false positives, further demonstrating its ability to detect mutations that real-time qPCR may miss. Moreover, we observed that certain samples were positive for double mutations, identified exclusively by ddPCR. Specifically, some samples initially positive for the −124C>T mutation also revealed the −146C>T mutation upon further analysis with ddPCR, highlighting ddPCR’s unique capacity for detecting low-frequency mutations with high sensitivity. This finding underscores the importance of further exploring these double positive samples, as they suggest a more intricate mutational landscape than previously recognized, potentially reflecting tumor heterogeneity or the presence of distinct clonal populations within the tumor. Such complexity in the mutation profile could have significant implications for understanding tumor biology, as tumors with heterogeneous or mixed clonal populations often exhibit more aggressive behavior and resistance to standard therapies.

Detecting *TERTp* mutations in urine samples offers significant clinical applications, particularly for bladder cancer management. This non-invasive approach allows for convenient monitoring of patients, enhancing compliance and comfort. Regular detection of these mutations can facilitate early identification of recurrences, potentially leading to timely interventions and improved outcomes [16,36]. Additionally, understanding the mutation profile may guide personalized treatment strategies and serve as prognostic indicators, helping oncologists assess disease aggressiveness. Overall, urine-based assays for *TERTp* mutations represent a promising advancement in oncology, with the potential to enhance patient management and inform therapeutic decisions.

The clinical significance of detecting low-frequency mutations using ddPCR is gaining recognition across various diseases. For instance, in non-small-cell lung cancer, 24% of clinically significant *EGFR* T790M resistance mutations were found at variant allele frequencies below 5%, underscoring the necessity for sensitive detection methods [37]. In Sturge–Weber syndrome, peptide nucleic acid ddPCR enabled the detection of low-prevalence *GNAQ* mutations in blood and saliva, suggesting its potential for non-invasive diagnostics [38]. While these studies illustrate the potential clinical relevance of low-frequency mutations across different conditions, larger-scale studies are essential to definitively establish their significance and determine appropriate fractional abundance thresholds for clinical decision-making.

The limitations of this study primarily stem from challenges associated with detecting low-frequency mutations in urine samples. While urine is a convenient, non-invasive sample source, the dilution of tumour-derived cells by normal epithelial cells complicates mutation detection. Although ddPCR showed improved sensitivity, the rain effect observed in some analyses complicated the separation of positive and negative results, affecting the clarity of mutation identification. Therefore, further optimization of droplet generation and reaction conditions is necessary to improve ddPCR accuracy. Additionally, the study’s limited sample size may restrict the generalizability of the findings, reducing statistical power in identifying mutation patterns across broader populations. As the first proof of concept for TERTmonitor in a ddPCR setting, this study demonstrates the assay’s potential to enhance mutation detection in urine samples. However, additional optimization is required, specifically regarding primer/probe concentrations and PCR conditions. Furthermore, DNA concentration data from samples show that many of the samples had low DNA input. Despite these low concentrations, ddPCR still detected mutations that real-time qPCR missed, emphasizing its robustness under suboptimal conditions. This highlights the need for even more refined approaches to improve the accuracy of both methods when working with limited DNA quantities.

Rigorous validation and optimization of ddPCR assays, including the primers/probes concentration and reaction conditions, are imperative to mitigate the impact of false positives associated with the rain effect. We propose that suboptimal sample quality, low target concentration, and inadequate analysis parameters may contribute to the observed rain effect [39,40,41]. Future investigations should prioritize refining these parameters to enhance ddPCR accuracy and reliability [39,42]. Despite these challenges, it is noteworthy that our analysis was still able to discern between negative and positive clusters. This underscores the robustness of the ddPCR method and highlights the potential for further optimization to mitigate the impact of contributing factors on data interpretation. Additionally, the spatial and temporal heterogeneity of bladder cancer adds complexity to mutation detection, complicating consistent capture across different tumour regions or time points [43,44].

Overall, this study demonstrated TERTmonitor’s suitability to be used either with real-time qPCR and ddPCR for detecting the −124C>T and −146C>T hotspot mutations, in a non-invasive diagnostic setting. While the use with ddPCR appears to provide increased sensitivity in capturing low-frequency mutations, further optimization will help reducing the “rain effect”, thereby enhancing ddPCR specificity and reliability. The choice between real-time qPCR and ddPCR should be guided by a comprehensive understanding of the specific mutations under investigation and the inherent limitations of each method.

## 5. Conclusions

In conclusion, our study aimed to address the potential use of TERTmonitor within a ddPCR framework, and for validation, we performed a comparative analysis alongside real-time qPCR. Our findings demonstrate similar performances of real-time qPCR and ddPCR in the detection of *TERTp* mutations. The high concordance observed across the majority of samples underscores TERTmonitor’s suitability to be used with both techniques. Noteworthy are the cases of exclusive positivity in ddPCR, hinting at a potential boost in sensitivity in certain situations, namely rain effect limitations. These findings highlight the importance of selecting the appropriate detection method based on the specific needs of the study, considering factors such as mutation frequency and sample characteristics. Furthermore, the observation of double mutations exclusively identified by ddPCR suggests a more complex mutational landscape, indicating the potential for tumour heterogeneity. To fully understand the clinical implications of these low-frequency mutations, particularly concerning fractional abundance thresholds, larger-scale studies are warranted. Continued research will be essential to optimize the ddPCR methodology and further establish the clinical significance of TERTmonitor in urogenital tumours and its possible incorporation in medical devices.

## Figures and Tables

**Table 1:** Comparative analysis of TERTmonitor mutation detection by real-time qPCR and ddPCR. Mutated (Mut) and wild-type (WT) samples for each methodology (real-time qPCR and ddPCR) underwent a direct head-to-head (H2H) comparison within the evaluated targets (−124 and −146). The comprehensive H2H comparison is summarised in two scenarios: “(+/+)” denoting concordant samples classified as Mut or as WT in both assays and “(−/+)”, indicating discordant samples, identified as WT by real-time qPCR and Mut by ddPCR; some samples were excluded from ddPCR analysis and therefore cannot be compared.

	−124			−146		
Sample	Real-Time qPCR	ddPCR	H2H	Real-Time qPCR	ddPCR	H2H
#1	**Mut**	**Mut**	(+/+)	WT	WT	(−/−)
#2	**Mut**	**Mut**	(+/+)	WT	WT	(−/−)
#3	**Mut**	**Mut**	(+/+)	WT	WT	(−/−)
#4	**Mut**	**Mut**	(+/+)	WT	WT	(−/−)
#5	**Mut**	**Mut**	(+/+)	-	-	-
#6	**Mut**	**Mut**	(+/+)	WT	WT	(−/−)
#7	**Mut**	**Mut**	(+/+)	WT	WT	(−/−)
#8	**Mut**	**Mut**	(+/+)	WT	WT	(−/−)
#9	**Mut**	**Mut**	(+/+)	WT	WT	(−/−)
#10	**Mut**	**Mut**	(+/+)	WT	WT	(−/−)
#11	**Mut**	**Mut**	(+/+)	WT	**Mut**	**(−/+)**
#12	**Mut**	**Mut**	(+/+)	WT	WT	(−/−)
#13	**Mut**	**Mut**	(+/+)	-	-	-
#14	-	-	-	WT	WT	(−/−)
#15	**Mut**	**Mut**	(+/+)	WT	WT	(−/−)
#16	**Mut**	**Mut**	(+/+)	WT	WT	(−/−)
#17	**Mut**	**Mut**	(+/+)	WT	**Mut**	**(−/+)**
#18	**Mut**	**Mut**	(+/+)	WT	WT	(−/−)
#19	**Mut**	**Mut**	(+/+)	**Mut**	**Mut**	(+/+)
#20	**Mut**	**Mut**	(+/+)	WT	WT	(−/−)
#21	**Mut**	**Mut**	(+/+)	WT	WT	(−/−)
#22	**Mut**	**Mut**	(+/+)	WT	WT	(−/−)
#23	**Mut**	**Mut**	(+/+)	WT	WT	(−/−)
#24	**Mut**	**Mut**	(+/+)	WT	WT	(−/−)
#25	**Mut**	**Mut**	(+/+)	WT	WT	(−/−)
#26	**Mut**	**Mut**	(+/+)	WT	WT	(−/−)
#27	**Mut**	**Mut**	(+/+)	WT	WT	(−/−)
#28	**Mut**	**Mut**	(+/+)	WT	WT	(−/−)
#29	**Mut**	**Mut**	(+/+)	-	-	-
#30	**Mut**	**Mut**	(+/+)	WT	**Mut**	**(−/+)**
#31	WT	WT	(−/−)	**Mut**	**Mut**	(+/+)
#32	WT	WT	(−/−)	**Mut**	**Mut**	(+/+)
#33	WT	WT	(−/−)	**Mut**	**Mut**	(+/+)
#34	WT	WT	(−/−)	**Mut**	**Mut**	(+/+)
#35	WT	WT	(−/−)	**Mut**	**Mut**	(+/+)
#36	WT	WT	(−/−)	**Mut**	**Mut**	(+/+)
#37	WT	WT	(−/−)	-	-	-
#38	WT	WT	(−/−)	WT	WT	(−/−)
#39	WT	WT	(−/−)	WT	WT	(−/−)
#40	WT	**Mut**	**(−/+)**	WT	WT	(−/−)
#41	WT	WT	(−/−)	WT	WT	(−/−)
#42	WT	**Mut**	**(−/+)**	WT	WT	(−/−)
#43	WT	WT	(−/−)	WT	WT	(−/−)
#44	WT	WT	(−/−)	WT	**Mut**	**(−/+)**
#45	WT	WT	(−/−)	-	-	-

## Data Availability

The original contributions presented in the study are included in the article/Supplementary Materials, further inquiries can be directed to the corresponding author.

## Supplementary Materials

The following supporting information can be downloaded at: www.mdpi.com/article/10.3390/genes15111424/s1, Table S1: Sample concentrations in nanograms per microliter (ng/µL), measured in a Nanodrop 1000; Table S2: TERTmonitor qPCR analysis of *TERTp* mutations; Table S3: TERTmonitor ddPCR analysis of *TERTp* mutations.
