# Supplementary material for: TERTmonitor Efficacy and Performance in Detecting Mutations by Droplet Digital PCR
Source: Genes (Basel). 2024 Oct 31;15(11):1424. doi: 10.3390/genes15111424 (PMC11594489; doi:10.3390/genes15111424)
Supplement: Supplementary file 1 [file genes-15-01424-s001.zip › genes-3281281-supplementary.pdf]

Supplementary materials

TERTmonitor efficacy and performance in detecting mutations by droplet digital PCR

Mafalda Bessa-Gonçalves [1,2], João Paulo Brás [1,2], Tito Teles Jesus [2], Hugo Prazeres [1,2], Paula Soares [1,2,3,4] and João Vinagre [1,2,3,4,*]

[1] U-Monitor Lda; 4200-135 Porto, Portugal
[2] Instituto de Investigação e Inovação em Saúde (i3S), Universidade do Porto, 4200-135 Porto, Portugal
[3] Instituto de Patologia e Imunologia Molecular da Universidade do Porto (Ipatimup), 4200-135 Porto, Portugal
[4] Faculdade de Medicina da Universidade do Porto (FMUP), 4200-319 Porto, Portugal

* Correspondence: jvinagre@ipatimup.pt; Tel.: +351225570700

Supplementary Table 1: Sample concentrations in nanograms per microliter (ng/µL), measured in a Nanodrop 1000.

Sample	*Concentration (ng/µL)*
#1	0,9
#2	4,3
#3	4,5
#4	3,9
#5	5,7
#6	2,8
#7	25,1
#8	7,6
#9	3,7
#10	10,1
#11	0,8
#12	11,5
#13	1,0
#14	18,7
#15	3,6
#16	2,9
#17	4,4
#18	5,4
#19	20,5
#20	4,6
#21	9,6
#22	7,2
#23	4,7
#24	5,6
#25	4,0
#26	9,0
#27	22,2
#28	9,4
#29	17,1
#30	3,3
#31	14,7
#32	35,1
#33	18,6
#34	0,7
#35	10,7
#36	39,3
#37	2,7
#38	6,2
#39	4,9
#40	2,6
#41	1,5
#42	3,0
#43	2,2
#44	4,9
#45	3,1

Supplementary Table 2: TERTmonitor qPCR analysis of *TERTp* mutations. qPCR data are presented in mutant (*Ct mut*) and wild-type Ct (*Ct wt*) values. Valid wild-type (WT) Ct values for this assay range between 25-40, while samples with *Ct mut* values below 45 are considered mutated (Mut). Samples with no amplification are labeled as NoA.

	- 124			- 146		
Sample	*Ct mut*	*Ct wt*	Result	*Ct mut*	*Ct wt*	Result
#1	36.84	33.11	Mut	NoA	33.94	WT
#2	31.42	30.62	Mut	NoA	30.53	WT
#3	32.62	30.23	Mut	NoA	31.65	WT
#4	30.23	32.96	Mut	NoA	33.75	WT
#5	32.79	28.46	Mut	NoA	28.61	WT
#6	31.88	30.10	Mut	NoA	30.35	WT
#7	32.95	30.75	Mut	NoA	31.97	WT
#8	35.76	34.36	Mut	NoA	31.29	WT
#9	41.11	32.82	Mut	NoA	33.25	WT
#10	38.99	29.81	Mut	NoA	30.50	WT
#11	35.39	35.47	Mut	NoA	36.49	WT
#12	35.18	32.55	Mut	NoA	32.91	WT
#13	35.46	32.48	Mut	NoA	34.73	WT
#14	30.94	27.90	Mut	NoA	28.93	WT
#15	42.97	29.63	Mut	NoA	31.62	WT
#16	30.80	29.64	Mut	NoA	31.30	WT
#17	34.79	33.36	Mut	NoA	33.20	WT
#18	31.97	29.00	Mut	NoA	30.44	WT
#19	33.82	31.66	Mut	37.17	31.96	Mut
#20	32.69	31.34	Mut	NoA	31.11	WT
#21	36.24	32.01	Mut	NoA	32.47	WT
#22	41.00	33.89	Mut	NoA	35.79	WT
#23	37.83	30.04	Mut	NoA	30.82	WT
#24	38.53	33.01	Mut	NoA	34.16	WT
#25	37.69	35.16	Mut	NoA	35.67	WT
#26	43.79	37.57	Mut	NoA	35.00	WT
#27	34.55	32.84	Mut	NoA	28.98	WT
#28	32.93	32.43	Mut	NoA	32.83	WT
#29	31.60	29.51	Mut	NoA	28.98	WT
#30	41.13	35.87	Mut	NoA	37.22	WT
#31	NoA	25.11	WT	29.86	27.35	Mut
#32	NoA	28.21	WT	35.47	30.05	Mut
#33	NoA	35.92	WT	38.45	34.49	Mut
#34	NoA	33.98	WT	40.19	35.53	Mut
#35	NoA	28.21	WT	29.92	30.03	Mut
#36	NoA	28.25	WT	31.97	26.96	Mut
#37	NoA	35.18	WT	40.08	35.32	Mut
#38	NoA	27.04	WT	NoA	29.13	WT
#39	NoA	23.56	WT	NoA	25.30	WT
#40	NoA	29.40	WT	NoA	31.18	WT
#41	NoA	31.36	WT	NoA	33.06	WT
#42	NoA	27.19	WT	NoA	28.34	WT
#43	NoA	37.49	WT	NoA	43.04	WT
#44	NoA	27.35	WT	NoA	29.71	WT
#45	NoA	28.09	WT	36.9	30.65	Mut

Supplementary Table 3: TERTmonitor ddPCR analysis of *TERTp* mutations. The outcomes are detailed concerning accepted droplets, encompassing positive mutated droplets ((+) mut), positive wild-type droplets ((+) wt), and fractional abundance (FA) for each assessed target (-124 and -146). Samples falling below a total of 10,000 accepted droplets were excluded (Exc). Those with FA values surpassing 1 were designated as mutated (Mut; result column), while samples exhibiting FA values below 1 were classified as wild-type (WT; result column).

	- 124					- 146				
Sample	Accepted droplets	*(+) mut*	*(+) wt*	FA	Result	Accepted droplets	*(+) mut*	*(+) wt*	FA	Result
#1	12476	3	17	14.99	Mut	17761	0	6	0.00	WT
#2	11793	245	488	33.19	Mut	16361	1	805	0.12	WT
#3	11938	32	192	14.20	Mut	16779	2	210	0.94	WT
#4	12446	9	63	12.48	Mut	16734	1	103	0.96	WT
#5	12121	133	542	19.43	Mut	4430	1	143	0.68	*Exc*
#6	17934	87	331	20.70	Mut	17364	0	36	0.00	WT
#7	15885	88	378	18.74	Mut	14888	0	435	0.00	WT
#8	16874	170	674	19.90	Mut	15951	0	728	0.00	WT
#9	16661	28	322	7.93	Mut	15312	1	342	0.29	WT
#10	15670	119	1634	6.47	Mut	14693	0	1651	0.00	WT
#11	16942	4	56	6.66	Mut	15436	12	42	22.21	Mut
#12	17145	47	236	16.53	Mut	15570	0	283	0.00	WT
#13	17695	26	177	12.76	Mut	2979	0	0	0.00	*Exc*
#14	2347	126	463	20.07	*Exc*	19612	5	5179	0.08	WT
#15	17679	107	491	17.73	Mut	13480	2	5179	0.39	WT
#16	18157	263	654	28.46	Mut	15143	5	917	0.53	WT
#17	10096	31	83	27.14	Mut	15785	4	207	1.88	Mut
#18	17526	203	1140	14.76	Mut	11461	0	818	0.00	WT
#19	16596	192	580	24.65	Mut	14907	37	754	4.57	Mut
#20	16861	287	560	33.70	Mut	13613	1	694	0.14	WT
#21	17307	63	201	23.79	Mut	15297	0	147	0.00	WT
#22	17257	5	61	7.56	Mut	17336	0	59	0.00	WT
#23	18247	94	829	10.00	Mut	14209	1	837	0.12	WT
#24	17218	29	142	16.91	Mut	16168	0	148	0.00	WT
#25	18238	27	95	22.10	Mut	17576	0	98	0.00	WT
#26	16846	13	44	22.79	Mut	16252	0	55	0.00	WT
#27	17369	344	1756	15.80	Mut	15991	1	2204	0.04	WT
#28	19524	14	360	3.71	Mut	10418	0	240	0.00	WT
#29	17659	403	1730	18.29	Mut	3156	1	457	0.20	*Exc*
#30	15817	34	33	50.75	Mut	16965	10	48	17.22	Mut
#31	15499	2	6685	0.02	WT	15748	1453	6245	16.08	Mut
#32	17119	1	1819	0.05	WT	15271	164	1604	8.87	Mut
#33	17558	0	79	0.00	WT	17424	9	63	12.48	Mut
#34	14553	0	38	0.00	WT	16830	5	53	8.61	Mut
#35	15401	3	1675	0.17	WT	15680	884	1247	41.19	Mut
#36	14825	2	4422	0.04	WT	13965	280	6230	3.31	Mut
#37	15357	0	61	0.00	WT	6886	1	15	6.24	*Exc*
#38	18263	2	3212	0.06	WT	16476	4	3458	0.10	WT
#39	17804	1	4328	0.02	WT	16685	2	4660	0.04	WT
#40	16474	8	210	3.65	Mut	16569	1	279	0.35	WT
#41	15334	0	27	0.00	WT	17233	0	56	0.00	WT
#42	15300	9	570	1.53	Mut	17413	3	510	0.58	WT
#43	14795	0	27	0.00	WT	16991	0	31	0.00	WT

**#44**	15210	0	644	0.00	WT	17762	17	877	1.86	**Mut**
**#45**	14534	0	804	0.00	WT	7440	11	104	9.51	*Exc*
